# OTDR Development Based on Single-Mode Fiber Fault Detection

**DOI:** 10.3390/s25144284

**Published:** 2025-07-09

**Authors:** Hui Liu, Tong Zhao, Mingjiang Zhang

**Affiliations:** 1 College of Physics and Optoelectronics, Taiyuan University of Technology, Taiyuan 030024, China; liuhui0212@link.tyut.edu.cn (H.L.); zhangmingjiang@tyut.edu.cn (M.Z.); 2 Key Laboratory of Advanced Transducers and Intelligent Control System, Ministry of Education, Taiyuan University of Technology, Taiyuan 030024, China; 3 Shanxi Key Laboratory of Precision Measurement Physics, Taiyuan University of Technology, Taiyuan 030024, China

**Keywords:** fiber fault monitoring, dynamic range, spatial resolution, chaotic OTDR, pulse-coded OTDR, PC-OTDR, LFM-OTDR

## Abstract

With the large-scale application and high-quality development demands of optical fiber cables, higher requirements have been placed on the corresponding measurement technologies. In recent years, optical fiber testing has played a crucial role in evaluating cable performance, as well as in the deployment, operation, maintenance, fault repair, and upgrade of optical networks. The Optical Time-Domain Reflectometer (OTDR) is a fiber fault diagnostic tool recommended by standards such as the International Telecommunication Union and the International Electrotechnical Commission. It is used to certify the performance of new fiber links and monitor the status of existing ones, detecting and locating fault events with advantages including simple operation, rapid response, and cost-effectiveness. First, this paper introduces the working principle and system architecture of OTDR, along with a brief discussion of its performance evaluation metrics. Next, a comprehensive review of improved OTDR technologies and systems is provided, categorizing different performance enhancement methods, including the enhanced measurement distance with simple structure and low cost in 2024, and the high spatial resolution measurement of optical fiber reflection events and non-reflection events in 2025. Finally, the development trends and future research directions of OTDR are outlined, aiming to achieve the development of low-cost, high-performance OTDR systems.

## 1. Introduction

Optical fiber cables, serving as a critical medium for information transmission, have been pervasively integrated into diverse societal domains. With the comprehensive deployment of new infrastructure initiatives—including 5G networks, data centers, the Industrial Internet, the Internet of Things, and artificial intelligence—the demand for real-time condition monitoring and performance evaluation of optical fiber cables has become increasingly pivotal [1]. Optical Time-Domain Reflectometry (OTDR), as a tool for diagnosing fiber faults, with the advantages of simple operation, rapid response, and low cost [2,3,4,5], has received extensive attention and favor from researchers and various manufacturers.

The OTDR system operates by injecting optical pulses into the fiber under test (FUT), and analyzing the attenuation characteristics along the fiber link through the intensity and temporal information of backscattered (Rayleigh scattering) and reflected signals. This methodology enables precise localization of fault events, including bending, fusion splices, mechanical connectors, and fiber breaks—all of which induce the power loss of the probe signal [6,7]. Among OTDR performance metrics, dynamic range and spatial resolution, which represent the long test distance and fault event identification capability, respectively, are the most critical parameters for evaluating system performance. Conventional optimization strategies involve broadening the probe pulse width and increasing peak power to extend the measurement range [8]. However, increased peak power intensifies nonlinear optical phenomena within the fiber, notably stimulated Raman scattering (SRS), stimulated Brillouin scattering (SBS), self-phase modulation (SPM), and four-wave mixing (FWM). Consequently, the maximum permissible pulse power is constrained by the nonlinear threshold to prevent signal distortion and measurement inaccuracies. Furthermore, pulse width adjustment presents certain limitations. While increasing the pulse width enhances the backscattered signal intensity and extends the dynamic range, it inevitably degrades the spatial resolution—a fundamental limitation governed by the positioning principle of the pulsed time-of-flight method.

Therefore, researchers have proposed various new schemes to solve the above limitations, such as the pulse encoding method, single-photon-counting method, chaotic correlation method, OTDR signal processing algorithm, etc. This manuscript will systematically review the physical principles, technical characteristics, and the latest research progress of the above schemes, and focus on analyzing the performance of each method in terms of breakthrough in dynamic range, improvement of spatial resolution, and optimization of measurement accuracy.

## 2. Theoretical Basis

### 2.1. Theoretical Model

**Figure 1:**
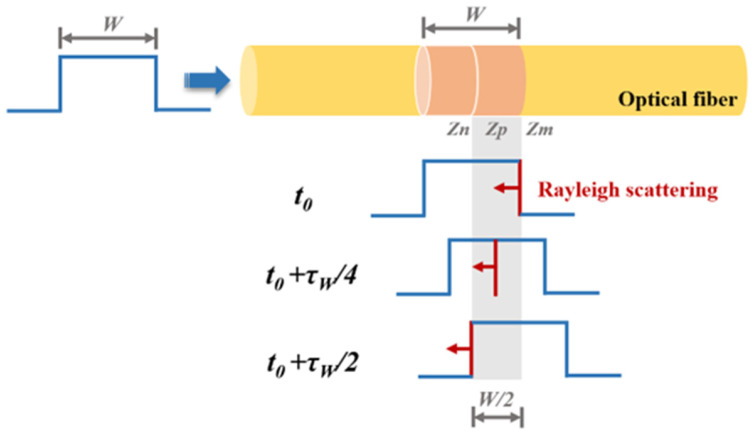
The theoretical model of OTDR.

The theoretical model of OTDR is schematically illustrated in Figure 1. An optical pulse with temporal width *τ_W_* (corresponding to spatial width *W*) is launched into the FUT. At time *t*_0_, the leading edge of the pulse reaches the fiber position *z_m_*, generating Rayleigh scattered light *Rz_m_*. During the process of *Rz_m_* returning to the starting position of the FUT, it encounters and combines with the Rayleigh scattered lights generated by the excitation of the pulse at other positions, and returns to the starting position of the FUT together. For example, at time *t*_0_ + *τ_W_*/4, *Rz_m_* arrives at the fiber position *z_p_*, where the propagating pulse simultaneously generates backscattered Rayleigh light *Rz_p_*. These two scattered lights (*Rz_m_* and *Rz_p_*) then co-propagate back toward the detection system. Similarly, when *t*_0_ + *τ_W_*/2 is reached, both *Rz_m_* and *Rz_p_* arrive at position *z_n_*, where the trailing edge of the pulse excites additional backscattered Rayleigh light. This newly generated light combines with the existing *Rz_m_* and *Rz_p_*, forming a composite backward-propagating signal. As the pulse continues propagating beyond *z_m_*, newly generated scattered lights cease to overlap with *Rz_m_*. Consequently, the detected backscattered signal ultimately comprises only those generated between the fiber positions *z_n_* and *z_m_*. This demonstrates that the Rayleigh scattered signal received at the detection system when the pulse reaches *z_m_* does not originate exclusively from *z_m_*, but rather from the superposition of all backward-scattered lights generated within a spatial interval corresponding to half the pulse width (*W*/2). The backscattered light generated at different times can combine at the same location because the incident pulse has a certain spatial width, and the backscattered signals generated by the leading and trailing edges of the pulse at different times converge during the transmission process. The above analysis indicates that when a pulse light with spatial width *W* is injected into an optical fiber, only the Rayleigh backscattered light generated within the range of *W*/2 can encounter each other and simultaneously reach the starting position of the optical fiber. This range is called the effective scattering zone, which determines the spatial resolution of the OTDR.

### 2.2. Schematic Architecture

**Figure 2:**
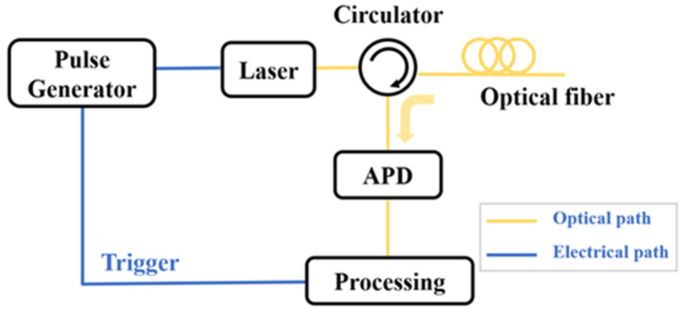
The schematic diagram OTDR system.

The schematic architecture of a typical OTDR system is illustrated in Figure 2 [9,10]. A pulse generator produces electrical driving signals that modulate a laser source, generating synchronized optical pulses. The pulse period is determined by the length of FUT and ensures that only one pulse can be transmitted in the optical fiber. These optical pulses are subsequently directed into the FUT through an optical circulator. The backscattered signals along the optical fiber are converted into electrical signals by an avalanche photodiode (APD) and then enter the data processing unit for analysis. The pulse generator needs to be triggered by the data processing unit to maintain the synchronization of the data. By detecting the intensity variations in the backscattered signals and Fresnel reflection signals along the FUT, the attenuation information and the length of the FUT can be measured; meanwhile, the loss and fault events can also be determined.

### 2.3. Typical OTDR Curve

**Figure 3:**
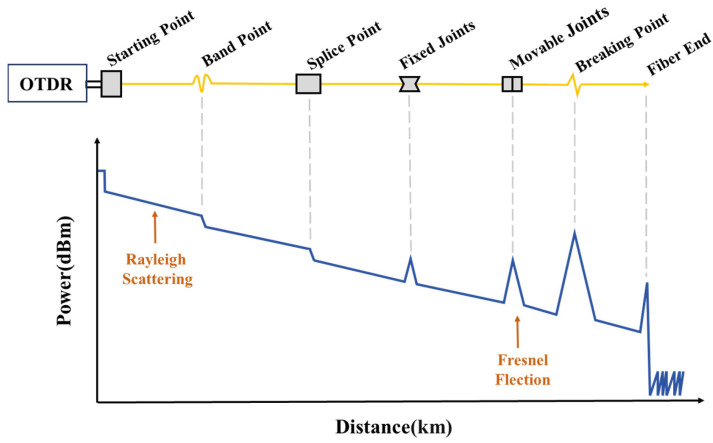
Typical OTDR test curve.

A typical OTDR test curve is shown in Figure 3. The horizontal axis represents the length of the FUT, while the vertical axis indicates the variation in optical power caused by different factors along the fiber link during the propagation of the probe pulse. If the fiber link operates normally, the OTDR trace exhibits a uniform attenuation trend, allowing the determination of the attenuation coefficient of the fiber. However, if an anomaly exists in the fiber link, the OTDR curve will exhibit abrupt changes.

Anomalies in fiber links primarily include reflective events and non-reflective events. Reflective events, caused by Fresnel reflections, manifest as sharp upward spikes in the OTDR trace, indicating potential issues such as connector mismatch, damaged mechanical splices, or fiber breaks. Non-reflective events refer to attenuation discontinuities in the OTDR curve, where the backscattered optical power suddenly drops at a specific position, accompanied by a change in the attenuation slope. These events appear as downward dips in the trace, suggesting possible issues such as degraded fusion splices or fiber bends. Both reflective and non-reflective events are generally referred to as fault events in optical fibers. Regardless of their type, such fault events can adversely affect the normal operation of fiber-based communication or sensing systems.

## 3. Performance Metrics

### 3.1. Dynamic Range

**Figure 4:**
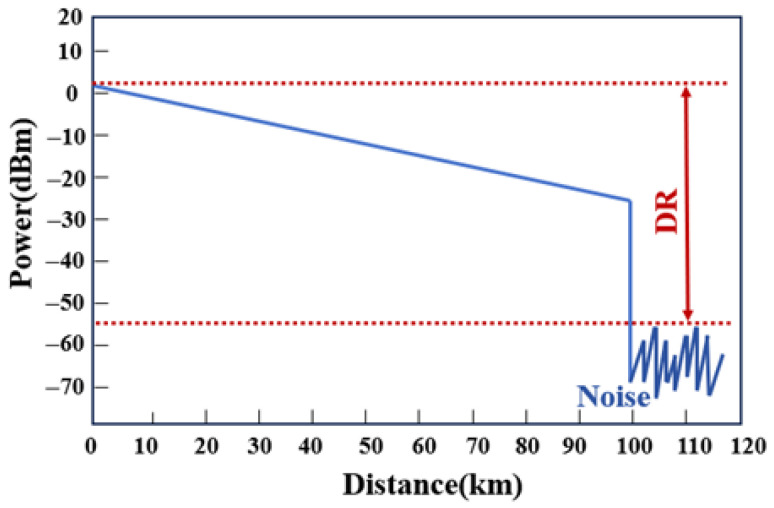
The diagram of dynamic range.

Dynamic range (DR) represents one of the most critical performance metrics for OTDR systems, fundamentally determining the maximum detectable distance of Rayleigh backscattering signals. Quantitatively, the dynamic range is defined as the power difference (in dB) between the initial backscattered peak power and the average noise floor at the end of the measurement trace, as illustrated in Figure 4.

The dynamic range can be defined as the ratio between the maximum backscattered light power received at the input end of the FUT and the system sensitivity [11]:

*DR* = [10 *lg*(*P_r_*/*P_s_*)]/2 = 5 *lg*(*P_p_ τ S*/*P_s_*) (1)

Here, 1/2 is the multiple factor, considering the round-trip process of the probe pulse through the forward transmission and backward return to the starting position of the FUT; *P_p_* is the peak power of the probe pulse, *τ* is the width of the probe pulse, *S* is the factor of Rayleigh backscattering of the fiber, and the system sensitivity *P_s_* represents the minimum detectable optical signal intensity at the receiver. A higher sensitivity implies that the OTDR system can detect weaker signals over longer distances and can be characterized by the noise-equivalent power (NEP) of the detector [12]:

*P_s_* ≈ *NEP(B/N)*^1/2^ (2)

where *B* denotes the detector bandwidth and *N* represents the number of averaging measurements in the system. Substituting (2) into (1) yields

*DR* = 5 *lg*{*P_p_ τ S*/[*NEP*(*B*/*N*)^1/2^]} (3)

As can be seen from Equation (3), the dynamic range of OTDR can be enhanced by increasing the peak power of the input optical pulse, extending the pulse width, or improving the system sensitivity.

### 3.2. Spatial Resolution

Spatial resolution is another critical parameter for evaluating OTDR system performance, representing the minimum distinguishable distance between two adjacent events, which can be expressed as [12,13]

*δz* = *v_g_ τ*/2 (4)

where *v_g_* denotes the speed of light in the optical fiber, and *τ* represents the pulse width.

Equation (4) indicates that the spatial resolution of OTDR is determined by the pulse width and exhibits an inverse proportionality to it.

### 3.3. Improvement Progress

**Figure 5:**
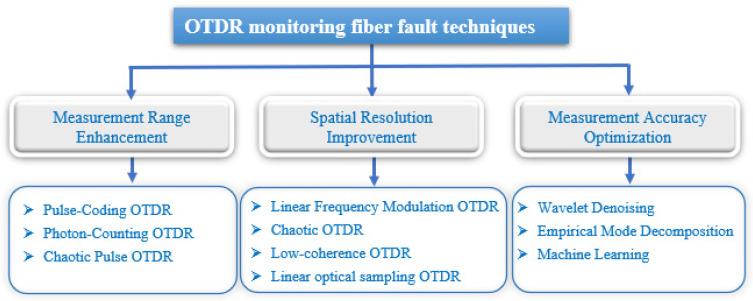
OTDR monitoring fiber fault techniques classification.

The advancement of OTDR systems has primarily focused on enhancing measurement range, improving spatial resolution, and optimizing testing accuracy. Figure 5 provides a classified summary of different improved OTDR techniques. In the following sections, we will discuss each technique in detail, presenting its working principle, advantages, and limitations.

## 4. Measurement Range Enhancement

### 4.1. Pulse-Coding OTDR

The fundamental principle involves increasing the number of optical pulses to enhance the energy injected into the fiber while keeping the pulse width unchanged. A coded pulse sequence composed of multiple pulses is launched into the fiber, and the backscattered signal at the receiver is decoded to obtain the loss distribution along the fiber, with the spatial resolution determined by the pulse width.

Compared to conventional single-pulse OTDR, multiple pulses enhance the optical pulse energy and the introduced coding gain improves the SNR, significantly reducing the required averaging time and number of averages. Consequently, this technique can also decrease the overall measurement time.

Over long-term development, pulse-coding schemes have evolved: from early pseudorandom codes that could not eliminate autocorrelation sidelobes [14], to Golay codes capable of achieving “complementary zero sidelobes” [15], followed by matrix-based Simplex codes (S-codes) [16], and eventually leading to hybrid coding schemes combining different code types [17].

#### 4.1.1. Pseudorandom Bit Sequences (PRBSs)

In 1980, K. Okada et al. [14] proposed the use of pseudorandom bit sequences (PRBSs) to modulate single pulses for enhancing the SNR of OTDR. Their experiments employed a light source with wavelengths ranging from 1 to 3 μm to detect a fiber breakpoint located at 10 km, demonstrating superior injected pulse energy and higher system SNR compared to conventional single-pulse OTDR.

M. Zoboli et al. [18] further demonstrated that increasing the length of the pseudorandom sequence could significantly improve the SNR of OTDR while achieving high-spatial-resolution measurements in 1983.

A systematic analysis of PRBS-based OTDR was conducted by J. K. A. Everard in 1987 [19]. This approach modulated the generated pseudorandom noise sequence onto the optical source before injection into the fiber, followed by cross-correlation processing between the received backscattered signal and a delayed version of the transmitted sequence to enhance the SNR of the system. Notably, this method required neither coherent detection nor special analysis. Compared with conventional OTDR systems, it increased the average transmitted and received signal power, thereby reducing either the required signal averaging time or the peak laser power while improving overall system performance.

However, in practical applications, the sidelobes inherent in the autocorrelation function of pseudorandom sequences introduce significant interference to OTDR measurements. While all aperiodic sequences exhibit some level of autocorrelation sidelobes, periodic sequences—despite the existence of sequences with zero sidelobes in their autocorrelation functions—present another challenge. The continuous transmission of periodic pulse trains causes prolonged saturation of the receiver, thereby severely limiting their engineering applicability.

#### 4.1.2. Golay Codes

In 1981, P. Healey [20] proposed that summing the correlation functions of specially designed sequences could reduce aperiodic correlation sidelobes or background noise to zero. This technique proved applicable to both unipolar and bipolar codes, with the generated truly orthogonal signals significantly enhancing the observation of Rayleigh backscattered light in optical fibers.

**Figure 6:**
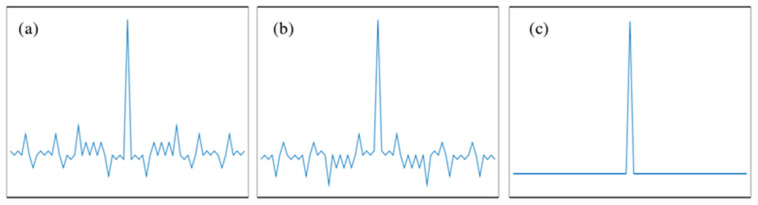
The aperiodic correlation functions of Golay codes. (**a**) Autocorrelation curve of A (32); (**b**) autocorrelation curve of B (32); (**c**) the sum of (**a**,**b**).

In 1989, M. Nazarathy and S. A. Newton et al. [15] formally introduced and experimentally demonstrated a complementary correlation technique based on Golay codes. Golay sequences consist of a pair of sequences A(k) and B(k) composed of −1 and 1, taking the 32 bit complementary sequences A (32) and B (32) as an example. Figure 6a and Figure 6b, respectively, show the shapes of their autocorrelation functions. It can be observed that both exhibit significant sidelobes, and their sidelobes are complementary. Figure 6c displays the sum of the two, where the sidelobes are completely eliminated. Unlike pseudorandom codes, Golay codes consist of two complementary sequences. Although the autocorrelation curve of each individual sequence contains sidelobes, their summation produces zero residual sidelobes.

Subsequent research has extensively investigated Golay code-based OTDR performance improvements through both numerical simulations and experimental validations [21,22,23,24,25]. In 2011, H. M. Liu [23] developed an embedded OTDR test platform employing 32 bit Golay codes with 40 ns pulse width. Through 4000 averaging iterations, the system achieved a single-pass dynamic range of 14 dB, representing a 7 dB improvement over conventional single-pulse OTDR under identical conditions. Further progress was demonstrated in 2016 by P. F. Wang [24], who implemented 32 bit Golay coding on probe pulses with 20 μs duration. After applying 150 kHz low-pass filtering, the OTDR system exhibited a 6 dB enhancement in dynamic range compared to traditional single-pulse implementations. A significant methodological advancement came in 2019 with R. L. Liao et al. [25], who proposed an oversampling technique for Golay-coded OTDR. This approach leverages oversampling to achieve additional coding gain, thereby further improving the SNR of the system.

#### 4.1.3. Orthogonal CCPONS Sequences

In 2008, P. K. Sahu et al. [26] proposed and experimentally validated a novel complementary correlation coding scheme—the Complementary Correlated Prometheus OrthoNormal Sequence (CCPONS). For four sets of CCPONS codes with code length L composed of “1” and “−1”, each complementary correlation sequence has an autocorrelation function peak magnitude equal to the code length L, along with sidelobes of certain magnitudes. However, the sum of the autocorrelation functions from all four complementary sequences equals 4L, with the sidelobes perfectly canceling each other out. All four complementary sequences contribute to the peak value, making the final results a collective outcome of all groups while effectively eliminating sidelobe interference. This serves as the theoretical foundation for applying CCPONS coding in OTDR systems. This coding scheme requires eight cycles to complete one measurement while providing higher SNR at the same code length. Their experimental results demonstrated that 64 bit CCPONS coding achieved a 3.7 dB improvement in unidirectional SNR compared to conventional OTDR under identical measurement conditions (20,000 averages and 100 mW peak power).

Further advancements were made in 2016 by D. Tian [27], who implemented 16 bit CCPONS coding with 30 ns pulse width in an OTDR system. After 1000 averaging iterations, the system exhibited a 7.8 dB enhancement in dynamic range over traditional single-pulse OTDR.

#### 4.1.4. Simplex Codes

In 1993, M. D. Jones [16] first theoretically proposed the application of Simplex Codes (S-codes) for enhancing OTDR dynamic range. A comprehensive comparison with Golay codes demonstrated that S-codes could achieve higher coding gain in OTDR systems.

This theoretical foundation was expanded in 2004 when D. Lee et al. [28] developed the fundamental algorithms and mathematical formulations for S-code implementation. The S-matrix is a unipolar matrix composed of 1 and 0, which can be derived from a normalized Hadamard matrix—the latter being a bipolar matrix consisting of 1 and −1. For a code length L, the coding gain is typically L, consistent with the result derived from the Hadamard transform spectrum. The experimental results verified a 4.5 dB improvement in single-pass dynamic range compared to conventional OTDR systems.

In 2006, D. Lee et al. [29] further validated the SNR enhancement capability of S-codes in OTDR systems. They proposed a universal formula for analyzing the performance of coded OTDR systems, establishing the relationship between coding gain and code length. Experimental results demonstrated a 2.3 dB SNR improvement compared to conventional OTDR when using 7 bit S-codes with a 10 μs pulse width, while increasing the code order to 255 with 0.5 μs pulses achieved a 9.2 dB SNR enhancement.

#### 4.1.5. Hybrid Coding Schemes

To achieve greater coding gain, in 2015, J. J. Liu et al. [30] developed an OTDR system based on CCPONS-Simplex composite codes, achieving SNR gain equal to the product of the individual gains from CCPONS codes and Simplex codes alone. The SNR enhancement could be further improved by increasing the sequence length of either coding scheme. Later in 2019, W. Cheng [31] introduced Golay-Simplex composite codes into OTDR systems, with simulation analysis demonstrating significant dynamic range improvement over single-pulse OTDR across various code lengths.

#### 4.1.6. Advantages and Limitations

The adoption of pulse-coding technology significantly improves signal anti-interference capability and enhances the SNR, enabling detection of remote events in fiber links. It only requires the introduction of encoding/decoding modules in the signal processing algorithm without modifying optical components (e.g., lasers, detectors), making it cost-effective.

However, it necessitates multiple transmissions of coded sequences and result accumulation, which extends measurement time. The decoding process involves matrix operations or iterative processing (such as Fast Hadamard Transform), imposing higher computational requirements on digital signal processing chips. Moreover, longer special code patterns are relatively more complex to encode and can also introduce certain difficulties in the decoding process.

### 4.2. Photon-Counting OTDR

Pulse coding optimizes the transmitted signal of the system, while single-photon detection enhances the performance at the receiver. Photon-counting OTDR (PC-OTDR) represents a high-sensitivity testing instrument that combines conventional OTDR technology with quantum detection techniques. Unlike traditional OTDR systems, PC-OTDR replaces linear-region photodetectors with single-photon detectors operating in Geiger mode [32]. Employing single-photon detection technology and time-correlated single-photon-counting methods enables the detection of extremely weak optical signals at the single-photon level, significantly enhancing weak-light detection capability and consequently improving the SNR. The discrete single-photon detection approach eliminates the limitation of spatial resolution by detector bandwidth and allows detection of ultra-low-power optical signals at the single-photon level. Furthermore, the performance improvement of the system is no longer constrained by the noise-equivalent power limitations of analog detectors [33,34,35].

#### 4.2.1. Evolution Process

With the development of optical fiber communication, the research on PC-OTDR has gradually focused on the long wavelength band. In 2002, F. Scholder et al. [36] experimentally obtained a dynamic range of 44 dB at a wavelength of 1550 nm. Compared with traditional OTDRs with the same spatial resolution, the dynamic range was increased by 4 dB. At the same time, a solution to suppress the disturbance dead zone was proposed. Using an intensity modulator with an extinction ratio of 30 dB in front of the detector to make the detector work in gated mode, the dead zone could be reduced by 15 dB.

In 2005, due to the extremely low photoelectric detection efficiency of Si-based single-photon avalanche diodes (Si-SPADs) in the near-infrared band, E. Diamanti et al. [37] introduced frequency up-conversion technology into the PC-OTDR system. They utilized the periodic polarization lithium niobate (PPLN) waveguide to complete frequency conversion from 1.5 μm to 0.8 μm, demonstrating superior performance compared to InGaAs/InP-APDs with significantly reduced dark count rates and post-pulse probability. This configuration eliminated the need for gated-mode operation while experimentally achieving 16 dB dynamic range with 1 m spatial resolution at the 1.5 μm communication wavelength. However, this scheme requires the use of quenching and reset circuits to switch the detection mode of Si-SPAD and reduce the dark count rate and post-pulse. This led to the need for several minutes to obtain the OTDR test curve, which limited the operating speed of the system.

**Figure 7:**
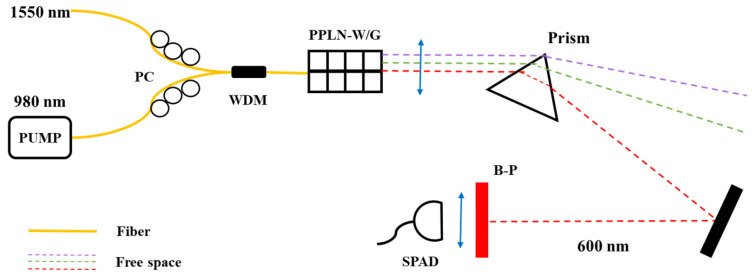
Setup of the photon-counting module based on sum frequency generation.

In 2007, M. Legre et al. [38] achieved centimeter-scale spatial resolution based on the up-conversion process in PPLN waveguides and low-time-jitter silicon photonic counting modules. The sum frequency generation (SFG) photon-counting module architecture is illustrated in Figure 7. This configuration utilizes SFG technology to combine a 1550 nm photon with a 980 nm photon, generating a 600 nm output photon. The upconverted light (600 nm) subsequently undergoes spectral filtering through a prism and interference filter before final detection by an SPAD.

With advancements in single-photon detection technology, PC-OTDR systems incorporating superconducting nanowire single-photon detectors (SNSPDs) have emerged as a prominent research focus [39,40,41]. In contrast to Geiger-mode APDs, SNSPDs operate under a constant bias current. They do not require a quenching circuit and have no after-pulses. They have the advantages of low time jitter, low dark count rate, and support for free mode, enabling the rapid acquisition of OTDR test curves.

In 2012, J. H. Hu et al. [11] proposed and experimentally validated a PC-OTDR system based on SNSPD. The system achieved a 22 dB dynamic range (corresponding to a 110 km sensing length) with a 15 min measurement time. It demonstrated a 6 cm spatial resolution at the 2 km position of an SMF and a 1.1 m resolution at the 26 km position. However, the practical implementation of SNSPD-based PC-OTDR systems remains constrained by several limitations, such as bulky detector size, the requirement for liquid nitrogen cooling, and prohibitively high costs, collectively restricting their widespread adoption.

In 2020, B. Li et al. [39] addressed the dynamic range limitation imposed by single-photon detector dead time through an externally time-gated PC-OTDR architecture. Their innovative approach employed temporal gating to restrict photon detection exclusively to the active window of the gate signal, effectively concentrating photon counts within the gated region rather than distributing them across the entire fiber. By synchronously scanning the gate signal, they achieved enhanced dynamic range measurements. Experimental results demonstrated an 11 dB dynamic range improvement compared to conventional PC-OTDR when using 50 ns gate pulses.

Based on this advancement, S. J. Deng et al. [42] developed a PC-OTDR-based fiber temperature sensor system in 2022. The system incorporated a newly developed high-speed photon-counting module enabled by an active quenching and reset integrated circuit, achieving a remarkable 3.8 cm spatial resolution over a 20 m FUT while maintaining rapid measurement capabilities.

#### 4.2.2. Engineering Implements

PC-OTDR systems have found extensive applications across multiple domains. Researchers at the University of Geneva implemented a gated PC-OTDR system for metro optical cable fault monitoring, achieving both high-speed and high-resolution fault localization [43]. At the Pontifical Catholic University of Rio de Janeiro, PC-OTDR technology was successfully deployed for real-time monitoring of optical communication networks, demonstrating superior resolution performance [44,45]. Researchers from the University of Electronic Science and Technology of China applied PC-OTDR to airborne avionics network monitoring, experimentally verifying the capability of the system for high-spatial-resolution testing of 32 core aircraft optical cables [46].

#### 4.2.3. Advantages and Limitations

The PC-OTDR employs highly sensitive single-photon detectors to further extend the dynamic range, while its discrete digital statistical approach enables spatial resolution to reach the centimeter level. However, the testing time is relatively long, as not every detection event captures a photon—a substantial accumulation of photon counts is required to accurately reconstruct the signal trace. This issue becomes particularly pronounced in long-distance fiber testing. Moreover, for a given PC-OTDR system, the detectable photon count is fixed. As the length of the FUT increases, the number of detectable photons per unit length decreases, which limits further improvement in dynamic range.

### 4.3. Chaotic-Pulse OTDR

**Figure 8:**
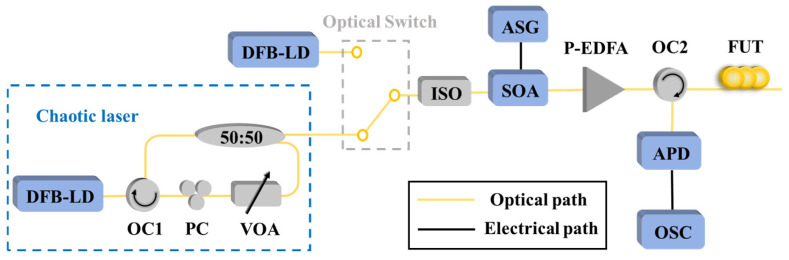
Setup of the OTDR based on chaotic-pulsed pulse. (DFB-LD: distributed feedback laser diode, PC: polarization controller, VOA: variable optical attenuator, SOA: semiconductor optical amplifier, P-EDFA: pulsed erbium-doped fiber amplifier, OC: optical circulator, APD: avalanche photodiode, ASG: arbitrary signal generator, OSC: oscilloscope; FUT: fiber under test).

In 2024, H. Liu et al. [47] improved the measurement distance of conventional OTDR by replacing traditional pulsed light with chaotic-pulsed light while maintaining the single-path detection structure and all other experimental conditions unchanged. The experimental setup is illustrated in Figure 8. The chaotic laser generated by the optical-feedback structure exhibits time-domain signals with random-fluctuation noise-like characteristics. After pulse modulation by the SOA, the high-level optical pulse carries the randomly fluctuating chaotic signals. A comparative analysis was conducted between traditional pulsed light and chaotic-pulsed light under identical conditions. The results demonstrate that, without any algorithmic processing, the chaotic laser enables a maximum measurement distance of up to 102 km—twice that of conventional light sources. This approach retains the simplest single-path detection structure of traditional OTDR, achieving a further enhancement in dynamic range without increasing system complexity or additional costs. However, compared to conventional continuous-wave light sources, chaotic sources in chaotic-pulse-based OTDR occupy a larger volume, which may result in greater scale requirements for commercial implementation.

It should be noted that this scheme is fundamentally different from chaotic OTDR [48]. Structurally, the proposed scheme employs only a single detection path without any reference path, and utilizes a chaotic-pulsed laser rather than a continuous-wave chaotic laser for detection. In principle, this approach detects fiber attenuation and fault locations by measuring Rayleigh backscattering signals. In contrast, chaotic OTDR can only determine fault positions through correlation operations and cannot obtain fiber attenuation information. X. Y. Dong et al. [49] proposed a joint measurement method based on chaotic OTDR for simultaneous attenuation and high-resolution fault detection. However, the essence of attenuation measurement still relies on the time-of-flight principle of OTDR, which does not deviate from conventional methods.

## 5. Spatial Resolution Improvement

### 5.1. Linear Frequency Modulation OTDR

**Figure 9:**
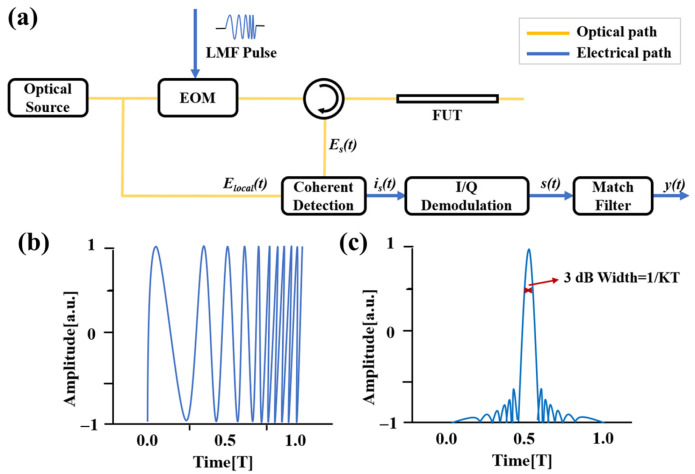
(**a**) OTDR schematic of pulse compression based on LFM technology; (**b**) time-domain shape of LFM pulse; (**c**) the compressed pulse with a 3 dB width of 1/KT.

To overcome the limitation of pulse width on spatial resolution and further improve the spatial resolution of OTDR systems, S. Yang et al. [50,51,52] proposed a novel pulse-compression OTDR based on linear frequency modulation (LFM) technology in 2014. The working principle is illustrated in Figure 9a. The optical source is split into two paths: one serves as the reference path, while the other is modulated with a pulsed LFM signal and injected into the FUT as the probe path. The backscattered signal from the FUT undergoes coherent detection with the reference signal. The electrical signal converted by the photodetector (PD) is then subjected to I/Q demodulation, and the backscattered curve is obtained through a match filter.

The original LFM pulse (Figure 9b) is compressed into a narrow sinc-shaped pulse (Figure 9c) during the pulse-compression process. As shown in Figure 9c, the full width at half maximum (FWHM or 3 dB width) of the main lobe is 1/KT, where KT represents the frequency modulation range. The spatial resolution is determined by the scanning range of the pulsed LFM rather than the pulse width, thereby resolving the trade-off between spatial resolution and dynamic range in conventional OTDR. Preliminary experimental validation of the pulse-compression OTDR was conducted. The system achieved a spatial resolution of 47 cm over a measurement range of 5.4 km, which is 2.7 times the coherence length of the light source.

**Figure 10:**
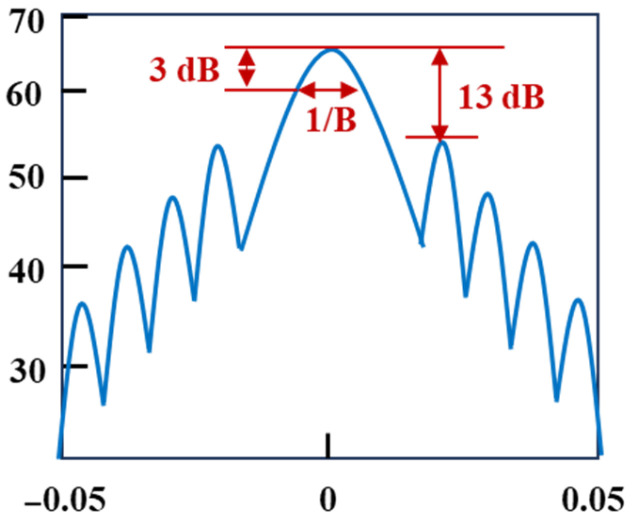
The principle of OTDR based on DLFM.

In 2019, P. Zhang et al. [53] proposed a digital linear frequency modulation (DLFM)-based OTDR that incorporated short-time fractional Fourier transform (STFrFT) for signal processing and noise filtering. The original LFM pulse was compressed into a narrow sinc-shaped pulse through STFrFT. As illustrated in Figure 10, the FWHM of the main lobe equals 1/B, where B represents the frequency modulation range.

By combining STFrFT with sidelobe suppression, both dynamic range and spatial resolution could be significantly enhanced simultaneously. The testing curve remained unaffected by laser phase noise, coherence length, or polarization state variations in backscattered light. Comparative experiments conducted on conventional OTDR development boards demonstrated that the proposed method achieved a 7 dB improvement in dynamic range and approximately a 10-fold enhancement in spatial resolution.

To further enhance the dynamic range and spatial resolution of LFM-OTDR, in 2020, Q. Wang et al. [54] proposed a novel approach for long-distance, high-precision OTDR using analog LFM signals with high-frequency modulation range and long pulse width. The proposed scheme employed an arbitrary waveform generator (AWG) and Mach-Zehnder modulator (MZM) to generate analog LFM signals with a 200 MHz frequency modulation range and 20 μs pulse width. Field testing was conducted on a 3 × 80 km fiber link in Tibet, achieving a spatial resolution of 53 cm. Compared to conventional single-pulse OTDR under identical conditions, this method demonstrated a significant 8.5 dB improvement in dynamic range.

**Figure 11:**
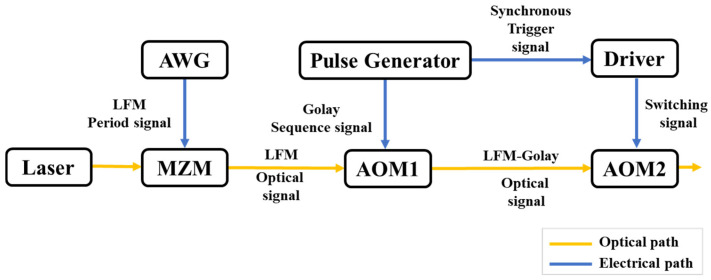
The principle of LFM-Golay OTDR.

In 2021, C. Liu et al. [55] proposed an innovative LFM-Golay OTDR, whose operational principle is illustrated in Figure 11. The system employs an AWG to produce LFM periodic signals with frequency characteristics that first linearly increase and then decrease within each cycle. These signals are subsequently converted into optical LFM signals through MZM. The Golay sequence signal generated by the pulse generator drives AOM1 to produce LFM-Golay optical signals, which are modulated into pulsed optical signals by AOM2 and injected into the FUT. Experimental results show that the LFM-Golay OTDR can achieve a spatial resolution of 0.22 m on a 200 km optical fiber link. Under the same test conditions, its dynamic range is 15 dB higher than that of the traditional single-pulse OTDR and 6 dB higher than that of the LFM-OTDR.

Expanding the signal bandwidth through linear frequency modulation enables centimeter-level or even millimeter-level resolution, significantly surpassing the performance of conventional pulsed OTDR. Continuous-wave modulation avoids high peak power pulses, thereby mitigating nonlinear effects such as stimulated Brillouin scattering. Frequency-domain processing suppresses noise and crosstalk, making it suitable for complex electromagnetic environments. However, this approach requires a high-precision linear frequency-modulated light source, a high-speed ADC, and sophisticated digital signal processing, resulting in higher hardware costs and development complexity. The linearity of frequency modulation, phase noise, and frequency drift directly impacts distance accuracy.

### 5.2. Chaotic OTDR

**Figure 12:**
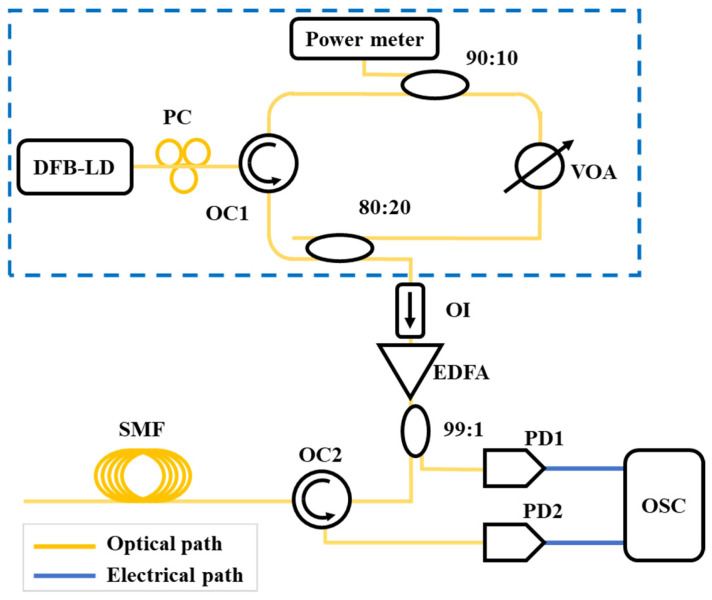
The principle of chaotic OTDR. (DFB-LD: distributed feedback laser diode, PC: polarization controller, VOA: variable optical attenuator, EDFA: erbium-doped fiber amplifier, OC: optical circulator, OI: optical isolator, PD: photodiode, OSC: oscilloscope; SMF: single-mode fiber).

Chaotic lasers have a wide spectrum and noise-like properties. The autocorrelation curve exhibits a δ-like function characteristic. The bandwidth of a chaotic laser can reach tens of GHz, resulting in an autocorrelation curve with a very narrow FWHM. When used in correlation detection, this enables high spatial resolution. Moreover, due to its noise-like chaotic waveform that does not repeat, it solves the problem of insufficient length in pseudorandom codes. Additionally, ordinary semiconductor lasers can generate chaotic waveforms without the need for expensive high-speed electronic devices for code generation, amplification, and modulation, making them suitable for large-scale applications. Therefore, Y. C. Wang et al. [48] introduced chaotic lasers into the OTDR system in 2008. They conducted proof-of-concept experiments that achieved 6 cm spatial resolution over a 140 m fiber link. The principle is shown in Figure 12. The location of the fault event is determined based on the time delay of the correlation peak. Unlike the traditional pulse-of-flight OTDR, the chaotic OTDR uses continuous light, and the spatial resolution is determined by the FWHM of the chaotic light autocorrelation curve. A higher bandwidth corresponds to a higher spatial resolution.

Building upon this foundation, chaotic OTDR has undergone a series of evolutions, primarily as follows.

#### 5.2.1. Generation Methods of Chaotic Laser

**Figure 13:**
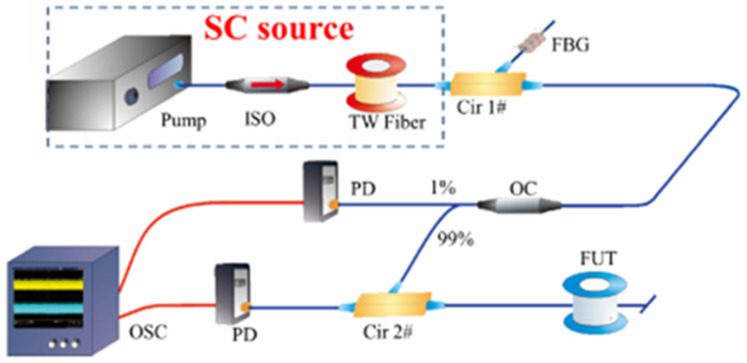
OTDR setup based on an all-fiber supercontinuum chaotic source.

In 2015, Z. N. Wang et al. [56] proposed a long-distance, high-precision correlation OTDR system based on an all-fiber supercontinuum chaotic source, with the experimental setup illustrated in Figure 13. The light source consisted of a 1455 nm Raman fiber laser coupled with a 16 km TrueWave (TW) fiber. By operating the pump laser in the anomalous dispersion regime of the TW fiber, modulation instability (MI) was induced to generate the supercontinuum source. This innovative configuration achieved a spatial resolution of 8.2 cm over a 100 km fiber.

**Figure 14:**
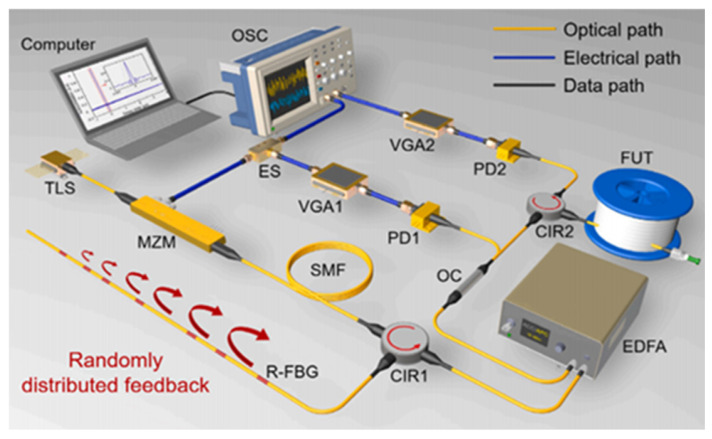
OTDR setup based on broadband random OEO.

In 2023, B. Li et al. [57] proposed and experimentally validated a novel correlation OTDR system based on a broadband random optoelectronic oscillator (OEO), as illustrated in Figure 14. Departing from conventional chaotic OTDR schemes that employ external-feedback semiconductor lasers as chaotic sources, this innovative approach utilizes a random OEO as the stochastic signal generator. With the help of the random distribution feedback caused by the random fiber Bragg grating, the generated random signal has a wide and flat spectrum and good autocorrelation characteristics. Experimental results show that this scheme can achieve a spatial resolution of approximately 10 mm independent of distance, and its dynamic range is at least 78.47 km.

In 2023, J. C. Li et al. [58] implemented a self-chaotic arc-edge microcavity laser with 12.9 GHz bandwidth and ±3 dB flatness in an OTDR system, achieving 4.5 mm distance resolution over a 25 km fiber link. This standalone broadband microcavity chaotic laser exhibits both broad spectral characteristics and aperiodicity, effectively eliminating the sidelobe interference typically observed in optical-feedback chaotic light correlation peaks, thereby significantly enhancing measurement accuracy.

#### 5.2.2. Chaotic Laser Integration

**Figure 15:**
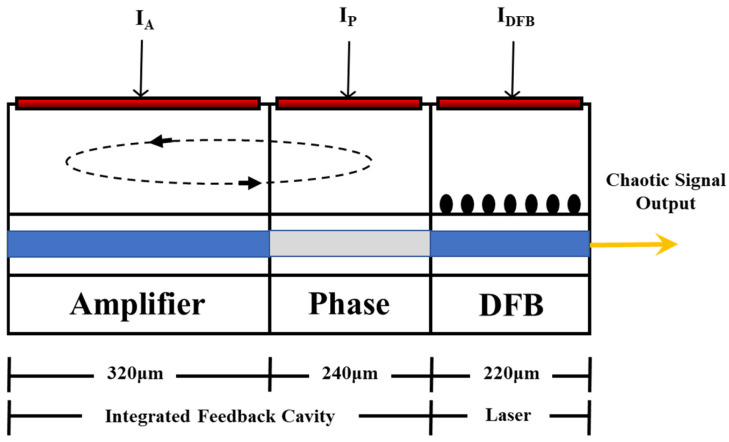
The structure diagram of MICL.

In 2017, L. M. Zhang et al. [59] proposed a compact, remote, and high-resolution chaotic correlation OTDR based on a monolithic integrated chaotic laser (MICL). MICL consists of a distributed feedback laser (DFB) section and a short-integrated feedback cavity, as shown in Figure 15. The 220 μm long DFB section serves as the laser source, while the integrated feedback cavity includes a 240 μm long phase section and a 320 μm-long amplifier section. The feedback phase and intensity can be controlled by injecting current. MICL can directly generate a broadband chaotic signal covering a frequency range of over 40 GHz. Using the OTDR based on MICL, the precise positioning of multiple reflection events was achieved, and a distance-independent spatial resolution of 2.6 mm was obtained on a 47 km optical fiber.

In 2019, M. W. Li et al. [60] developed a correlation OTDR technique based on hybrid-integrated short-external-cavity chaotic semiconductor lasers (SCSL). The hybrid-integrated SCSL generated chaotic signals exhibiting characteristic oscillations with random fluctuations in the time domain. The experiment achieved the measurement of multiple single-reflection events and double-reflection events, with a dynamic range of 76.54 km and a spatial resolution of 1 cm.

#### 5.2.3. Bandwidth Performance Enhancement

**Figure 16:**
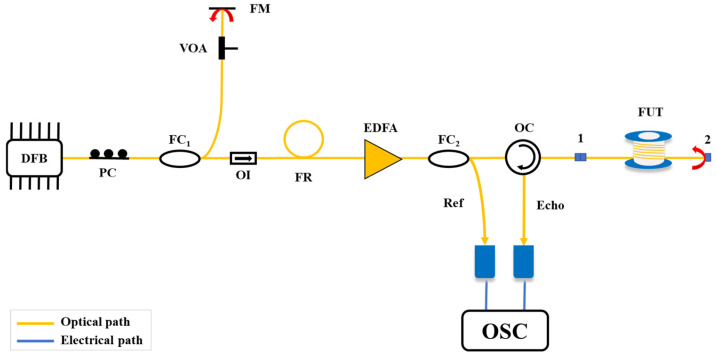
OTDR setup based on the chaotic light source incorporated a fiber loop.

In 2019, Z. H. Hu et al. [61] further optimized the chaotic light source by incorporating a fiber loop into the chaotic generation structure. As shown in Figure 16, this configuration enhanced the low-frequency component energy through multi-beam interference and delayed self-heterodyning effects. The improved system achieved fault localization over 49.4 km of optical fiber, demonstrating a 5 dB dynamic range enhancement compared to conventional chaotic OTDR systems without fiber loops.

#### 5.2.4. Laser Self-Responding

**Figure 17:**
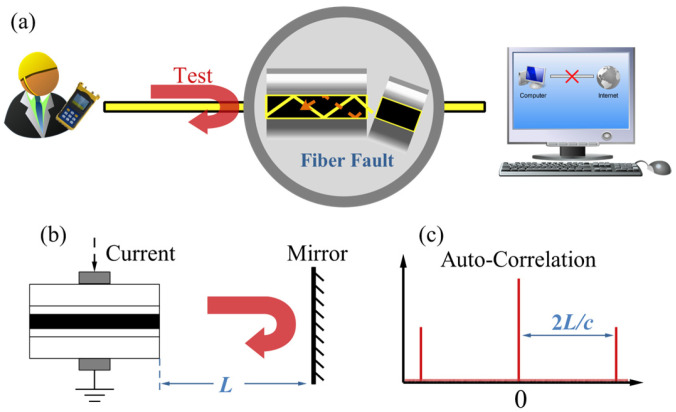
Principle of the monitoring method. (**a**) Fault detection depends on the reflection information. (**b**) Typical chaos generation structure with optical feedback. (**c**) Autocorrelation of the chaotic laser subject to optical feedback.

In 2015, T. Zhao et al. [62] proposed a fault detection method utilizing the time-delay characteristics of optical-feedback semiconductor lasers, shown in Figure 17. In this approach, the semiconductor laser serves as the detection light source. When feedback reflections from fiber faults occur, the laser enters a chaotic state. The autocorrelation of the output time series reveals external cavity characteristics, thereby indicating the distance between the laser and the fiber fault.

However, this method relies on extracting time-delay features from the chaotic output of the laser, which requires sufficient feedback strength (typically above −50 dB) to generate chaos. Consequently, when the reflected feedback from a fault point in the fiber falls below −50 dB, the corresponding time-delay signature can no longer be reliably extracted from the temporal waveform.

#### 5.2.5. Frequency Resonance

**Figure 18:**
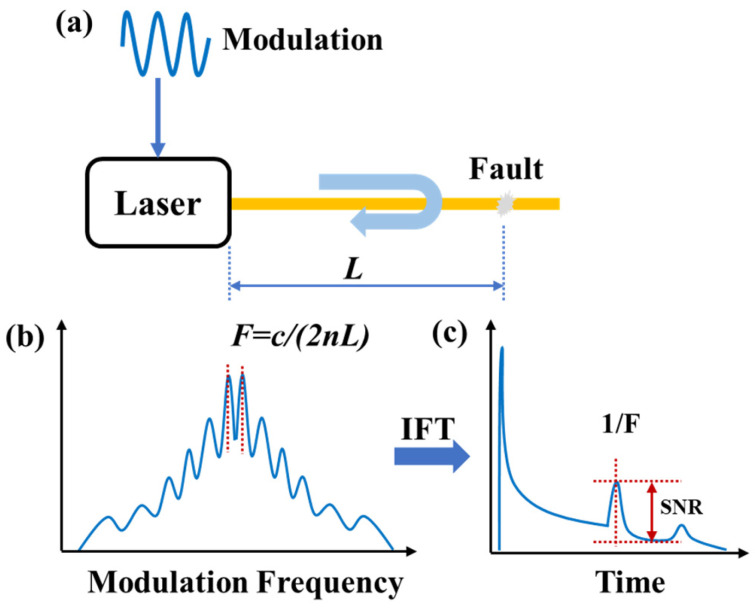
Schematic of fiber fault detection by frequency resonance method. (**a**) Setup: a modulated laser receiving its own delayed feedback from the fault point; (**b**) modulation response curve; and (**c**) its inverse Fourier transform.

In 2022, Z. X. Shi et al. [63] proposed a novel method for extracting time-delay signatures that is not constrained by the operational state of the laser. Its schematic is illustrated in Figure 18. The technique involves superimposing a frequency-swept current modulation signal onto the bias current of the laser. By recording the amplitude response of the laser to different modulation frequencies, the modulation response curve of the laser is obtained.

When the modulation frequency approaches the external cavity frequency or its integer multiples, a resonance phenomenon occurs between the modulation current and the external cavity, causing the response of the laser to reach a maximum. The feedback time-delay characteristics are then extracted from the modulation response curve of the semiconductor laser through inverse Fourier transform (IFT). Finally, the extracted time-delay signatures are used to locate fiber fault points.

#### 5.2.6. Joint Measurement of Fiber Loss and Attenuation

**Figure 19:**
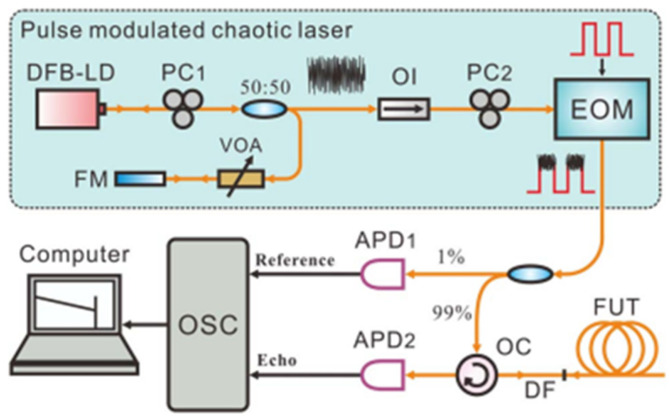
Experimental setup of the joint measurement.

While the above schemes can achieve long-distance, high-spatial-resolution fault detection, due to the use of continuous light as the probe light, it is impossible to effectively measure the backscattered signals at various positions in the optical fiber, and it is not possible to monitor the status of the optical fiber. To address this, in 2015, X. Y. Dong et al. [51] modulated a continuous chaotic laser into a chaotic-pulsed laser and divided it into two paths of light, shown in Figure 19. One path was used as the probe light to inject into the FUT, and the other path was used as the reference light to correlate with the backscattered signals along the optical fiber. The spatial resolution was still determined by the bandwidth of the chaotic laser, and the joint measurement of optical fiber attenuation information and high-resolution fault detection was achieved. Experimental results showed that this scheme achieved 10 km of optical fiber attenuation measurement. Due to the 200 MHz bandwidth of the photodetector, a spatial resolution of 0.6 m was obtained in a 5 km optical fiber.

In 2017, S. Q. Guo et al. [64] proposed a chaotic-pulse hybrid signal OTDR. Using continuous chaotic laser and pulse light to measure the Fresnel reflection events and Rayleigh scattered signals in the optical fiber, respectively, and linearly superimposing the results of the two measurements. The measurement curve along the optical fiber was obtained, solving the problem that chaotic correlation OTDR cannot measure optical fiber loss. The optical fiber loss of 50 km was experimentally measured, and a spatial resolution of 35 cm independent of distance was obtained within a range of 104 km.

#### 5.2.7. Measurement of Fiber Attenuation Event

OTDR systems that achieve high spatial resolution based on correlation methods have mainly focused on reflection events in optical fiber links. There have been few reports on the measurement of attenuation events such as bends and splices, whose specific location and length cannot be obtained through direct correlation operations. The reason is that the measurement object for reflection events is the echo signal at a certain position, which is equivalent to an amplified probe signal. After direct correlation processing, the specific location of the reflection event can be determined based on the time delay of the correlation peak. In contrast, the measurement object for attenuation events is a sudden reduction in the backscattered signal within a defined attenuation region of specific length. Moreover, the backscattered signal does not show the same random-fluctuation characteristics as the probe signal, making it impossible to directly use the correlation method to locate attenuation events.

To address these limitations, in 2025, H. Liu et al. [65] propose an OTDR technique based on Attenuation Extraction-Correlation Compression Integrative Demodulation (AECCID) that simultaneously achieves high spatial resolution and precise localization for measuring fiber attenuation faults. This approach employs a broadband chaotic laser as the light source and performs differential processing on the backscattered signals to extract loss information from attenuation fault regions. Through correlation analysis, the method accurately determines both the start and end positions of attenuation events. Simulations demonstrate successful measurements of both single and multiple events with a spatial resolution of approximately 8 mm. In experimental validation using a 1000 ns pulse width, limited by the detector bandwidth, a spatial resolution of 0.87 m was achieved.

#### 5.2.8. Advantages and Limitations

The bandwidth of chaotic signals can reach tens of GHz, enabling spatial resolution on the millimeter scale. Their noise-like characteristics provide strong anti-interference capability, allowing reliable operation in complex environments. However, compared to conventional continuous-wave light sources, chaotic light systems exhibit relatively complex structures and larger footprints. Moreover, the signal energy is distributed across a wide frequency band, resulting in low power spectral density at individual frequencies and a poor signal-to-noise ratio for long-range detection.

### 5.3. Low-Coherence OTDR

**Figure 20:**
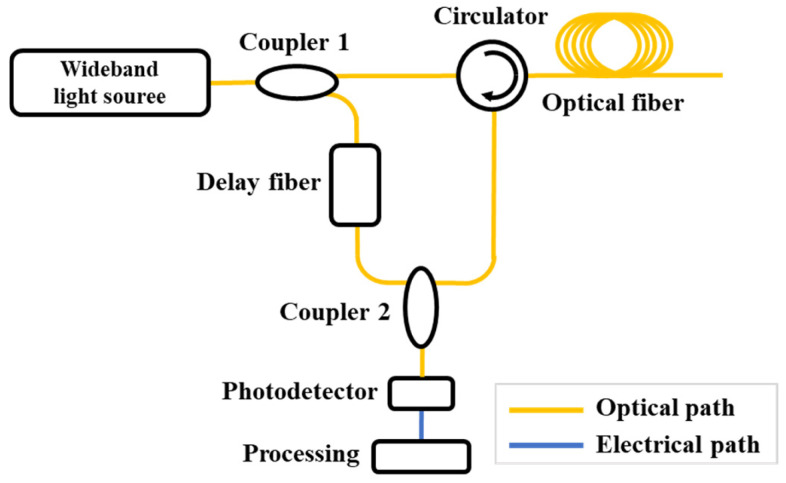
Experimental setup of the LC-OTDR.

Low-coherence OTDR (LC-OTDR) relies on broadband light sources such as superluminescent diodes (SLDs) or supercontinuum sources. These sources exhibit a wide spectral bandwidth and a short coherence length (typically on the order of micrometers to millimeters). The short coherence length ensures that significant interference occurs only when the optical path difference between two beams is extremely small. Consequently, the spatial resolution of LC-OTDR is determined by the spectral linewidth (or coherence length) of the light source, enabling high spatial resolution. A schematic of the LC-OTDR structure is illustrated in Figure 20.

The wideband light source is split by Coupler 1 into a probe beam and a reference beam. The probe beam is injected into the optical fiber via an optical circulator, and the backscattered Rayleigh signal or reflected signal returns to Coupler 2. Meanwhile, the reference beam passes through a tunable optical delay fiber before entering Coupler 2, where it interferes with the backscattered signal. Interference occurs only when the optical path lengths of the reference beam and the backscattered signal are matched. The resulting interference signal is converted into an electrical signal by a photodetector and then processed (e.g., smoothing, filtering) by a data acquisition and analysis system to extract the characteristics of the fiber. By adjusting the length of the tunable optical delay fiber in the reference path, distributed measurements along the entire fiber can be achieved.

Low-coherence OTDR (LC-OTDR), as a high-performance optical fiber sensing technique, demonstrates outstanding capabilities in high resolution and sensitivity. However, it suffers from several inherent limitations—including coherence-induced noise, stringent source requirements, system complexity, restricted measurement speed, polarization dependence, limited dynamic range, and constrained applicability—which hinder its further development.

### 5.4. Linear Optical Sampling OTDR

**Figure 21:**
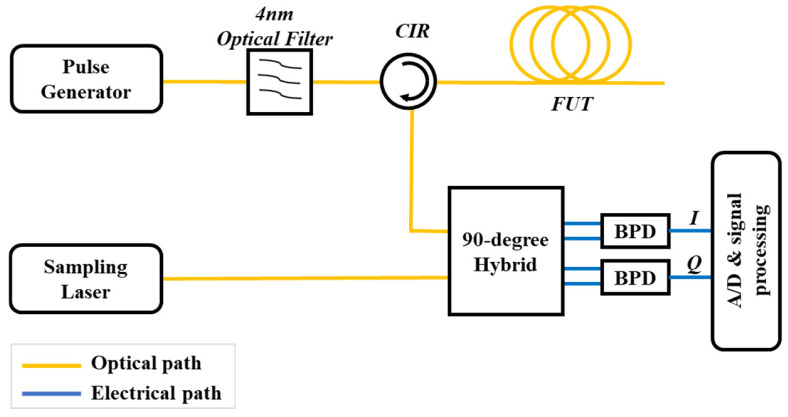
Experimental setup of the optical sampling OTDR.

In 2017, Z. Y. He et al. [66] proposed an ultrahigh-resolution OTDR system employing a mode-locked laser as the pulse source combined with linear optical sampling technology, whose operating principle is illustrated in Figure 21. Ultrashort optical pulses are generated by a pulse generator and injected into the FUT through an optical circulator. The backscattered signal from the fiber is directed to the third port of the circulator. Simultaneously, a sampling laser emits short pulses with a repetition rate synchronized to the pulse generator, which interfere with the backscattered signal. The resulting interference signal is detected by a balanced photodetector (BPD) and subsequently processed through A/D conversion.

As the measurement distance increases, the pulse width broadens due to dispersion effects and the wide bandwidth of the ultrashort pulses. By implementing digital dispersion compensation techniques, the system achieved a spatial resolution of 340 μm when measuring a reflector at a 10 km distance. The optical sampling approach primarily relies on extended integration time to enhance performance, effectively trading measurement duration for bandwidth requirements. This method enables the acquisition of higher signal power while maintaining resolution. Furthermore, the incorporation of high-sensitivity photodetectors facilitates a substantially improved dynamic range.

Linear optical sampling OTDR utilizing ultrashort pulse lasers and coherent detection can achieve picosecond-level temporal resolution, millimeter-scale spatial resolution, and a dynamic range exceeding 50 dB. However, this technique presents challenges including high system complexity, substantial hardware costs, stringent requirements on source stability, and the necessity for high-performance FPGA/GPU processors to handle real-time processing of massive data volumes.

## 6. Measurement Accuracy Optimization

The SNR of OTDR traces directly determines the measurement accuracy of the system while simultaneously being a critical factor constraining other performance metrics. When SNR is low, the backscattered signal from the fiber end becomes indistinguishable from noise, limiting the maximum detectable fiber length. Moreover, scattering signals carrying fault information may be completely obscured by noise, significantly impacting fault detection and identification. Traditional averaging represents the most conventional noise reduction approach [67], which operates by accumulating and averaging multiple measurement traces—while useful signal amplitudes remain relatively stable, random noise averages toward zero through multiple accumulations. However, this method becomes impractical for large datasets as single measurements become excessively time-consuming, compromising timely optical network maintenance. Subsequent research has proposed wavelet transforms, empirical mode decomposition (EMD), and deep learning-based interference detection methods, all of which enhance OTDR system SNR to varying degrees through software/algorithmic optimizations.

### 6.1. Wavelet Denoising

In 2006, L. R. Tang et al. [68] proposed a wavelet transform-based method to enhance fault detection accuracy in OTDR systems, where high-frequency components from wavelet decomposition correspond to abrupt signal variations, and applying threshold filtering and signal enhancement processing to these high-frequency components effectively suppresses noise while amplifying information at transition points. Simulation results demonstrated that this algorithm maintains high accuracy and reliability even under strong noise interference conditions.

In 2010, A. R. Bahrampour et al. [69] proposed a Fourier-Wavelet Regularized Deconvolution (ForWaRD) method based on an adaptive wavelet approach, which enhances the SNR of the system since deconvolution is inherently noise-sensitive. Through simulations of long-pulse OTDR systems, they demonstrated that combining wide input laser pulse widths with the ForWaRD method could improve the dynamic range of OTDR while achieving centimeter-level spatial resolution.

In 2013, L. H. Yu et al. [70] developed an improved undecimated discrete wavelet transform (UDWT) denoising technique that more effectively separates signals from noise through undecimated sampling and multi-resolution analysis, demonstrating superior performance in OTDR applications compared to conventional discrete wavelet transform (DWT) by preserving complete signal information without any loss during processing.

In 2019, H. M. Peng et al. [71] processed the OTDR curve using a filtering algorithm based on wavelet transform. They introduced a special threshold processing algorithm, which retained the characteristics of the hard threshold method without changing the prominent signal feature, while also considering the continuity feature of the soft threshold method. After denoising processing, the OTDR curve was not deteriorated and could better preserve the edges of the reflection events. In the same year, L. Zhou et al. [72] conducted two-level denoising on the OTDR signal. They applied the wavelet threshold method for the first-level denoising and performed the second-level denoising by matching the characteristic signals in the real-time data. They also compared this method with the traditional cumulative averaging method. The experiments proved that this method could effectively reduce the detection noise while shortening the testing time and improving the positioning accuracy of fault events.

Wavelet denoising effectively separates noise (high-frequency components) from useful signals (low-frequency components) through soft- or hard-thresholding of wavelet coefficients, enabling adaptive noise suppression. However, its performance heavily depends on the empirical selection of wavelet bases (e.g., Daubechies, Symlets), with different bases significantly influencing denoising outcomes. The method is also highly sensitive to threshold selection: excessive thresholds may oversmooth meaningful signal features, while insufficient thresholds retain residual noise.

### 6.2. Empirical Mode Decomposition

In 2007, Z. G. Qu [73] proposed an EMD-based feature extraction technique for detection signals. The decomposed intrinsic mode functions (IMFs) represent stationary signals at characteristic scales, successfully applying kurtosis—a parameter sensitive to pulse signals—as the characteristic feature of vibration signals for leakage detection and early warning in distributed optical fiber monitoring of long-distance oil and gas pipelines.

In 2015, recognizing that OTDR signals contain non-stationary noise and events can be treated as singularities, Q. Han et al. [74] developed a hybrid algorithm combining EMD and wavelet transform that first employs EMD for signal denoising and then utilizes wavelet transform for singularity detection, with experimental results demonstrating its effectiveness in accurately detecting and locating OTDR events, showing significant practical application value.

In 2017, H. Hao et al. [75] proposed a novel multivariate denoising scheme based on multivariate empirical mode decomposition (MEMD). Different from previous EMD-based denoising methods, it aligns common frequency modes across multiple channels of multivariate data, thereby enabling direct multichannel data denoising.

In 2020, Q. Han et al. [76] developed a denoising algorithm combining variational mode decomposition (VMD) and singular spectrum analysis (SSA). OTDR signals are first decomposed into multiple intrinsic mode functions using VMD. Each component is then denoised through SSA processing, and finally the denoised components are reconstructed to obtain the enhanced OTDR signal. Experimental results demonstrate that this VMD-SSA hybrid approach significantly improves the SNR of OTDR measurements.

Empirical Mode Decomposition (EMD) adaptively decomposes signals into Intrinsic Mode Functions (IMFs) without requiring predefined basis functions, thereby better preserving localized signal transients. However, signals from different physical processes (e.g., noise overlapping with weak reflection events) may become mixed within the same IMF, leading to incomplete denoising. The method necessitates iterative IMF sifting, resulting in computational complexity significantly higher than wavelet transforms, which limits its practicality for real-time processing of large-scale OTDR datasets.

### 6.3. Machine Learning

In 2021, Z. M. Yang et al. [77] addressed the low detection sensitivity of conventional OTDR event identification methods by developing a machine learning-based detection approach. Firstly, the OTDR signal is subjected to n-order differential processing and denoised. Then, it identifies and analyzes peak characteristics in the differentiated signals to extract features for offline classifier training, with the trained model subsequently deployed for online event prediction. This algorithm has been verified through 500 OTDR traces and the results show that the detection rate of connection splicing events is as high as 95%.

In the same year, K. Abdelli et al. [78] proposed a novel multi-task learning model based on long short-term memory (LSTM) to detect, locate, and estimate the reflectivity of reflective fiber events, including connectors and mechanical splices. It extracts insights from monitoring data obtained through OTDR measurements, which are commonly used for optical cable or link fault diagnosis. Experimental results demonstrated that the proposed method achieves favorable detection capability and high localization accuracy even under low SNR conditions while requiring shorter measurement times. That same year, the authors introduced a data-driven approach based on convolutional neural networks (CNNs) to detect and characterize reflective fiber faults in noisy simulated OTDR data with SNRs ranging from 0 dB to 30 dB, including reflective event patterns. Compared with conventionally employed techniques, the proposed method achieved superior localization accuracy under lower SNR conditions while maintaining a lower false alarm rate.

In 2022, addressing the limitations of existing machine learning models under low signal-to-noise ratio (SNR < 10 dB) conditions—where their generalization and robustness capabilities may severely degrade their adaptability and effectiveness in responding to new, unknown data—K. Abdelli et al. [79] proposed a novel approach combining a denoising convolutional autoencoder (DCAE) with a bidirectional long short-term memory (BiLSTM) network. The DCAE was employed for noise reduction in OTDR signals, while the BiLSTM was utilized for fault detection, localization, and diagnosis using the denoised signals as input. This method was applied to noisy OTDR signals with input SNRs ranging from −5 dB to 15 dB. Experimental results demonstrated that the DCAE effectively denoised OTDR signals, outperforming other deep learning techniques and conventional denoising methods. Furthermore, the BiLSTM achieved a fault detection and diagnosis accuracy of 96.7%, representing a 13.74% improvement compared to the same model trained on noisy OTDR signals.

The machine learning autonomously learns complex features without requiring predefined filtering parameters. However, its performance depends on extensive labeled datasets for training, while acquiring field data entails significant costs.

### 6.4. Trend Filtering

In 2015, G. C. Amaral et al. [80] proposed and demonstrated an automatic optical fiber fault analysis system utilizing a tunable PC-OTDR, which was experimentally validated on a passive optical network test platform. By employing an *ℓ*1 trend filter as the signal processing tool, the system effectively mitigated the impact of inherent coherent random noise on the measurement data while enabling automatic fiber fault identification. This filtering approach can also be extended to few-mode fibers [81].

It is worth noting that numerous methods have been developed to enhance measurement accuracy. For instance, in 2019, M. Zabihi et al. [82] proposed a multi-frequency detection-based φ-OTDR system, achieving distortion-free output signals with over 98.85% fidelity. In 2008, K. Suh et al. [83] employed two pulsed laser sources with a wavelength difference equal to twice the Raman frequency shift. By alternately injecting these pulses into the sensing fiber via an optical switch, they compensated for temperature measurement inaccuracies caused by inconsistent Raman attenuation coefficients. However, these methods primarily aim to improve temperature or strain measurement performance, differing from the OTDR-based fiber fault detection approach presented in this manuscript.

## 7. Monitoring Required Features and Techniques Comparison

An OTDR instrument that meets the requirements for engineering applications must satisfy multiple criteria. We have summarized and elaborated on common application needs, and conducted a comparative analysis of different monitoring techniques, as presented in Table 1.

(1) Fault Detection: In OTDR systems, fault detection refers to the process of identifying, locating, and characterizing anomalies (e.g., fiber breaks, bends, splices, or connector losses) in an optical fiber link by analyzing backscattered and reflected light signals.
(2) Cost: The cost of OTDR equipment is a critical consideration in engineering applications and system deployment. As a key instrument for optical fiber network testing and maintenance, its cost-effectiveness directly impacts several aspects, such as large-scale deployment feasibility, operation and maintenance expenses, and return on investment.
(3) Complexity: The structural complexity of OTDRs can severely constrain their engineering applications. Such limitations directly conflict with core industry demands for cost-effective, compact, and field-serviceable OTDRs—particularly in distributed fiber sensing or telecom network monitoring where reliability and efficiency are critical.
(4) Reliability: The system possesses a stable operational capability to achieve specified objectives while meeting expected performance metrics during repeated operations.
(5) Notification Time: The time from fault occurrence to system response. Shorter durations indicate higher system response rates, enabling faster repairs.
(6) Automatic: Network operators are capable of gathering monitoring information and identifying failures without the need for on-site technical personnel deployment.
(7) Deployed: The monitoring technology should be applicable to deployed networks without requiring any modifications to the network infrastructure.
(8) Scalability: The adaptability of the monitoring technique to evolving network infrastructure configurations.

**Table 1:** Advantages and limitations of different monitoring techniques.

Advantages and Limitations		Fault Detection	Cost	Complexity	Reliability	Notification Time	Automatic	Deployed	Scalability	Engineering Application
**Monitoring** **Techniques**										
Measurement Range Enhancement	Pulse-Coding OTDR	Y	M	M	Y	M	Y	Y	Y	Submarine Optical Cable
	Photon-Counting OTDR	Y	H	L	Y	Lo	Y	Y	Y	Quantum Key Distribution Link Monitoring
	Chaotic-Pulse OTDR	Y	L	L	Y	S	Y	Y	Y	Submarine Optical Cable
Spatial Resolution Improvement	LFM-OTDR	Y	M	M	Y	S	Y	Y	Y	Aircraft Wing Bending
	Chaotic OTDR	Y	L	L	Y	S	Y	Y	Y	High-Speed Railway Catenary Systems
	Low-coherence OTDR	Y	H	M	Y	Lo	Y	Y	Y	Structural Monitoring of OCT Catheters
	Linear Optical Sampling OTDR	Y	H	H	Y	Lo	Y	Y	Y	Connector Insertion Loss in Data Center Fiber Patch Cords
Measurement Accuracy Optimization	Wavelet Denoising	Y	L	L	Y	S	Y	Y	Y	Aging Fiber
	EMD-based OTDR	Y	L	L	Y	M	Y	Y	Y	Composite Cables (Fiber + Copper)
	ML-based OTDR	Y	L	L	Y	Lo	Y	Y	Y	Intelligent Operation and Maintenance

Y: Yes; N: No; H: High; M: Medium; L: Low; Lo: Long; S: Short.

As summarized in Table 1, the applicable scenarios for different monitoring technologies can be clearly defined as follows.

(1) Pulse-coding OTDR: This technique is particularly suited for detecting minute losses in long-haul trunk fibers (e.g., inter-city/regional backbone networks, submarine cables) where high dynamic range is required but single-pulse power cannot be increased.
(2) Photon-counting OTDR: This technique is ideally suited for low-light-intensity scenarios, such as detecting weak backscattered signals in optical fiber sensor networks or performing fault localization in quantum communication fiber links.
(3) Chaotic-pulse OTDR: This technique is particularly applicable to cost-sensitive scenarios where fault spacing is large and millimeter-level precision is not required.
(4) LFM-OTDR: This technique is particularly suitable for applications requiring high localization accuracy, such as rapid and precise fault identification in fiber-to-the-home (FTTH) networks and exact position detection of optical fiber sensors in industrial automation systems.
(5) Chaotic OTDR: In complex electromagnetic environments, chaotic OTDR exhibits superior anti-interference capability, effectively resisting external electromagnetic disturbances and malicious attacks, thereby ensuring the stable operation of optical fiber communication systems. This technique is particularly employed for safeguarding critical communication infrastructure.
(6) Low-coherence OTDR: The technique is suitable for fault localization scenarios where high spatial resolution is prioritized over strict requirements for detection distance.
(7) Linear optical sampling OTDR: This technique is particularly suitable for remote optical identification and diagnostics in passive optical network (PON) links, as well as for precise fault localization in aircraft systems.
(8) Wavelet-denoised OTDR: Wavelet-denoised OTDR is particularly effective in noisy optical fiber environments, such as aging fiber infrastructure or complex industrial settings, where it can efficiently suppress noise while extracting weak fault signatures, thereby significantly improving fault detection accuracy.
(9) EMD-based OTDR: This method is particularly applicable for multi-scale fault analysis in non-uniform fiber links, including hybrid fault localization in composite cables (fiber + copper) and long-term monitoring of diverse coupling losses (bending, splicing, aging).
(10) ML-based OTDR: This approach is particularly suitable for intelligent operation and maintenance management in optical fiber communication networks, significantly enhancing fault-handling efficiency. It enables the prediction of future loss trends in optical fiber links and evaluation of different maintenance strategies, thereby providing critical decision support for network planning and optimization.

It should be noted that in recent years, with the increasing demand for communication capacity, novel optical fibers such as hollow-core fibers [84,85,86], few-mode fibers [87,88,89,90,91], and multi-core fibers [92] have emerged. Taking few-mode fibers as an example, they support multiple transmission modes, each exhibiting unique propagation characteristics and mutual interactions, resulting in distinct differences in fault detection compared to single-mode fibers. Consequently, researchers have investigated the backscattering principles in few-mode fibers. Leveraging the higher fault detection sensitivity of higher-order mode backscattered Rayleigh light, they proposed methods utilizing higher-order modes to achieve comprehensive evaluation and precise localization of loss events in few-mode fibers. However, these fault detection techniques are not applicable to traditional single-mode long-haul communication fibers, and thus, they will not be elaborated in detail in this manuscript.

## 8. Conclusions and Future Prospects

### 8.1. Conclusions

As an essential tool for optical fiber fault diagnosis, OTDR plays a pivotal role in evaluating optical cable performance and supporting the deployment, operation, maintenance, fault repair, and upgrade of optical networks. This paper introduces the developmental background and physical principles of OTDR, elaborating on its evolution in the field of optical fiber fault detection. Significant advancements have been achieved in expanding dynamic range, such as low-cost long-distance chaotic-pulse OTDR in 2024; improving spatial resolution, such as high-resolution measurements of non-reflective events in optical fibers at the centimeter and even millimeter scale in 2025; and enhancing measurement accuracy. These improvements have not only strengthened the capability of OTDR in detecting faults within complex fiber links, but also broadened its application scope across diverse optical networks. Through algorithmic optimization and hardware upgrades, OTDR can now pinpoint fiber breaks with higher precision and assess connection losses with greater reliability, thereby providing robust support for network stability. Furthermore, the continuous maturation of OTDR technology has laid a solid foundation for the overall advancement of optical fiber communication systems.

### 8.2. Technological Bottlenecks

Despite significant advancements in OTDR performance enhancement, several challenges remain to be addressed, as follows:

(1) Trade-off between dynamic range and spatial resolution. Increasing the pulse width enhances the input pulse energy, thereby improving the OTDR dynamic range. However, this simultaneously degrades the spatial resolution of the OTDR system.
(2) Real-time performance versus computational complexity. High-precision algorithms (e.g., wavelet denoising, machine learning) require extensive computations, making real-time monitoring challenging.
(3) Cost-performance trade-off. High-performance OTDR systems are cost-prohibitive, while low-cost devices exhibit limited capabilities, making them unsuitable for all application scenarios.

### 8.3. Future Prospects

(1) Cost Reduction. Currently, the high cost of high-performance OTDR equipment limits its widespread application in small-scale optical networks. Optimizing hardware design and manufacturing processes to reduce costs while improving device usability and operability will facilitate broader adoption of OTDR technology.
(2) System Integration. The integration of OTDR functionality into optical network units demonstrates significant advantages in terms of fault detection efficiency and operational expenditure reduction.
(3) Intelligent Processing. The deep integration of OTDR technology with emerging technologies such as AI, big data, and the IoT represents a key future development trend. By enabling intelligent acquisition, transmission, storage, and analysis of OTDR test data, more efficient, intelligent, and automated optical fiber fault detection and maintenance systems can be established.

## Data Availability

No new data were created or analyzed in this study.
